# Doing housework and having regular daily routine standing out as factors associate with physical function in the older people

**DOI:** 10.3389/fpubh.2023.1281291

**Published:** 2023-11-28

**Authors:** RuiQi Li, YaLun Dai, YiWen Han, Chi Zhang, Jing Pang, Jian Li, TieMei Zhang, Ping Zeng

**Affiliations:** ^1^The Key Laboratory of Geriatric Medicine, Beijing Institute of Geriatrics, Institute of Geriatric Medicine, Chinese Academy of Medical Sciences, Beijing Hospital/National Center of Gerontology of National Health Commission, Beijing, China; ^2^Graduate School of Chinese Academy of Medical Sciences and Peking Union Medical College, Beijing, China

**Keywords:** physical function, gait speed, Geriatric Depression Scale, mini-mental state examination, doing housework, lifestyle

## Abstract

**Background and objectives:**

Nationwide data were used to explore factors associated with physical function in order to identify interventions that could improve and maintain physical function in the older people.

**Methods:**

The physical function was assessed by gait speed (GS). We selected 2,677 male and 2,668 female older adults (aged ≥60) who could perform the GS test as study subjects. GS was measured by having subjects walk across and back a 10-m course. A gait speed less than 20% that of a reference population (<0.7 m/s) was used as the definition of slow gait speed (SGS). Co-morbidity, polypharmacy, medical expenses, need for care, and hospitalization were used to evaluate health status. A stepwise logistic regression model was used to determine factors associated with SGS.

**Results:**

SGS was associated with poorer health status, higher medical cost, lower ranking on the Geriatric Depression Scale (GDS) and decreased Mini-mental State Examination (MMSE). Co-morbidity (*OR* = 1.81, 1.58–2.07), polypharmacy (*OR* = 1.47, 1.25–1.74), MMSE <24 (*OR* = 1.85, 1.54–2.22), and GDS ≥ 11 (*OR* = 1.40, 1.18–1.65) were associated with SGS. In contrast, doing housework (DHW, *OR* = 0.43, 0.38–0.49), having a regular daily routine (RDR, *OR* = 0.64, 0.45–0.91), and current alcohol consumption (*OR* = 0.74, 0.62–0.90) were inversely associated with SGS. DHW plus having RDR could greatly reduce the risk of SGS (*OR* = 0.29, 0.19–0.43).

**Conclusion:**

Poor physical function is associated with poorer health status in Chinese older people. Maintaining a regular daily routine and doing some housework may be important factors that can help older people preserve their physical function.

## Background

Population aging has underscored the importance of successful aging, which involves maintaining physical and mental function to enable older people to live independently until the end of their lives (1). Factors contributing to physical and mental well-being in older people include race, gender, age, education attainment, amount and type of physical activity, chronic diseases, and adaptation to physical and emotional stressors (2, 3). Physical well-being has been emphasized for since it facilitates active life engagement, social participation, and promotes self-efficacy (2). The contribution of physical activities to better physical function has been widely acknowledged. In contrast to the protective effect of a healthy lifestyle, the presence of diseases and poor mental and psychological status are risk factors for the reduction of physical function in older people (3, 4). These studies suggest that multimodal strategies, such as disease intervention, physical activity, and cognitive stimulation are effective in maintaining physical function.

Since aging is a comprehensive and complex process, the individual’s own motivation to respond to the challenges of aging should also be encouraged (5). Ng et al. adapted the model of successful aging to Chinese cultural context, and suggested that aging well socially through engagement with life and doing or helping with housework being a part of daily life engagement (6). The older people’s motivation respond to the challenges of aging may be reflected in their daily life engagement and routine (6). However, these factors have not been fully explored as means of preserving physical function in older people (5, 7). Walking requires considerable energy and coordination, and walking speed may reflect the multi-systemic well-being of older people. The speed of walking is closely related to the strength of the body, balance and endurance. So, using gait speed (GS) as an indicator of physical function, this study explores factors, in addition to well-recognized ones, that are associated with slow gait speed (SGS) to generate intervention strategies in the population, especially among those who are still ambulatory but may be at risk of a reduction in physical function and loss of independence.

## Methods

### Participants

The data for this study were obtained from a nationwide cross-sectional survey conducted in 2011 that evaluated the health status of older Chinese. The study was conducted by investigators and participants from 13 hospitals in six administrative regions of China (North China, Southwest China, Northwest China, Central China, East China, Northeast China). The study subjects were community residents and patients in regular health examination centers, outpatient departments, and wards of the participating hospitals. The following criteria were used for subjects selection: (1) age ≥ 60 years; (2) ability to successfully complete physical function tests, such as the gait speed assessment; (3) provision of oral informed consent. Subjects with severe cognitive impairment, which hindered their ability to cooperate with the investigation, were excluded from the analysis.

### Studied variables

Questionnaires were used to gather information on demographics, lifestyle, and medical history, including receipt of care, co-morbidity, polypharmacy, medical expenses, increased need for care and hospitalizations. Co-morbidity was defined as having two or more common diseases, such as hypertension, diabetes, cardiovascular disease, coronary heart disease, chronic obstructive pulmonary disease (COPD), osteoarthritis, and cancer. Polypharmacy was defined as currently taking more than five kinds of medicines. Cognitive function was evaluated by the Mini-mental State Examination (MMSE) (8). Psychological status was measured by the Geriatric Depression Scale (GDS) (9), which included the expression of dissatisfaction with health and disinterest in life. Doing housework (DHW), considered as an indicator of daily life engagement and independence, was defined as a person doing work such as house cleaning, cooking, and taking care of grandchildren whose frequency was self-described as “often”; i.e., at least 5 days a week. Having a regular daily routine (RDR) was defined as a person having a regular daily schedule of activities. The lifestyle data collected in the study included currently smoking (≥5 cigarettes/day), regular consumption of alcohol (at least once a week), and engaging in physical exercise (PE, moderate or more, at least once a week and lasting for 30 min or more).

Before the survey was administered, the interviewers were trained, and the subjects’ oral informed consent was obtained to use their information in this study. The Ethics Committee of Beijing Hospital, Ministry of Health, approved the study.

### Measurement

The physical function was assessed by GS, which was evaluated by measuring the time for the subject to walk 20 m. Participants walked at their usual pace from a standing start and continued walking straight forward for 10 meters, at which point they made a U-turn and returned to the starting line. SGS was defined as a value in the 20th percentile of GS measured in the participants in this study, which was <0.7 m/s.

### Statistical analysis

Descriptive statistics, such as means, standard deviations, and proportions, were used to characterize the demographics and measured variables of the subjects. The differences in continuous variables were compared using analysis of variance with two factors (GS and gender). Frequency data were compared by Cochran–Mantel–Haenszel statistics to remove confounding influences. Stepwise logistic regression was performed (Model 1) to estimate the adjusted odds ratios and the 95% confidence interval (95%*CI*) of the variables associated with SGS. Using predicted probabilities, receiver-operating characteristic (ROC) curves were constructed to evaluate the discriminative ability of the model (Model 1). The area under the ROC curve (AUC) was used to test the concordance of predictive values with actual outcomes. The clustering of MMSE<24 and/or GDS ≥ 11 (clustering number 0 = none, 1 = having either one, 2 = having both factors) and the clustering DHW and/or RDR (clustering number 0 = none, 1 = having either one, 2 = having both factors) with the risk of SGS were further studied by logistic regression (Model 2). The statistical analyses were carried out using SAS software and *p* < 0.05 was considered statistically significant.

## Results

The study had a total of 5,345 participants, with 2,677 men and 2,668 women. The health status and characteristics of the subjects with normal GS (NGS) and SGS are shown in Table 1. The mean age of the population was about 70 years, with the SGS group being about 3 years older than the mean of the NGS group, but this difference was not statistically significant. The difference in the mean value of GS for each group was statistically significant, with the mean of the SGS group being 0.5 m/s slower than that of the NGS group.

**Table 1:** The health status and characteristics of the older people with normal and slow gait speed.

Variables	Normal gait speed		Slow gait speed		*P1*	*P2*
	M (1,443)	F (1,530)	M (1,234)	F (1,138)		
Age	70.88 ± 7.41	68.91 ± 6.56	73.70 ± 8.09	71.46 ± 7.52	0.1216	<0.0001
Education ≥12 years (%)	614 (42.3)	806 (52.7)	603 (48.9)	703 (61.8)	<0.0001	<0.0001
BMI (kg/m^2^)	24.09 ± 3.37	24.09 ± 3.45	24.00 ± 3.36	24.24 ± 3.55	0.7564	0.2589
BMI ≥ 25 kg/m^2^ (%)	515 (35.7)	437 (35.4)	536 (35.0)	428 (37.6)	0.405	0.342
Gait speed (m/s)	1.04 ± 0.28	1.00 ± 0.25	0.47 ± 0.14	0.48 ± 0.14	<0.0001	0.0037
Co-morbidity (%)	457 (31.7)	575 (37.6)	705 (57.1)	600 (52.7)	<0.0001	<0.0001
Polypharmacy (%)	230 (15.9)	225 (14.7)	389 (31.5)	276 (24.3)	<0.0001	<0.0001
Medical cost (RMB)	4,210 ± 1,000	3,196 ± 1,000	9,778 ± 2000	5,893 ± 1,500	<0.0001	<0.0001
Care demanding (%)	258 (18.0)	235 (15.4)	371 (30.3)	302 (26.7)	<0.0001	0.0064
Hospitalization (%)	351 (24.4)	335 (22.0)	528 (43.2)	388 (34.3)	<0.0001	<0.0001
Dissatisfied with health	117 (8.1)	195 (12.7)	220 (17.8)	219 (19.2)	<0.0001	0.002
MMSE scores	27.99 ± 3.09	27.02 ± 4.17	26.28 ± 4.13	25.42 ± 5.00	<0.0001	<0.0001
MMSE scores <24 (%)	104 (7.2)	227 (14.8)	227 (18.4)	307 (27.0)	<0.0001	<0.0001
GDS scores	4.57 ± 4.31	5.59 ± 5.01	6.53 ± 5.03	7.02 ± 5.41	<0.0001	<0.0001
GDS scores ≥11 (%)	147 (10.2)	244 (16.0)	278 (22.7)	287 (25.4)	<0.0001	<0.0001
Disinterested in life	23 (1.6)	23 (1.5)	27 (2.2)	32 (2.8)	<0.0001	0.610
Doing housework (%)	701 (48.6)	1,223 (79.9)	342 (27.7)	625 (54.9)	<0.0001	<0.0001
Regular daily routine (%)	605 (42.1)	657 (42.9)	384 (31.4)	400 (35.2)	<0.0001	0.0054
Current smoking (%)	445 (30.8)	28 (1.8)	315 (25.5)	32 (2.8)	0.0180	<0.0001
Current drinking (%)	396 (27.4)	54 (3.5)	244 (19.8)	41 (3.6)	<0.0001	<0.0001
No physical exercise (%)	307 (21.3)	328 (21.5)	310 (25.4)	266 (23.5)	<0.0001	0.01947

MMSE, Mini-mental State Examination; GDS, Geriatric Depression Scale. Current smoking:≥5 cigarettes/day. Current drinking: at least once a week. Physical exercise: moderate or more, at least once a week and lasting for30 min or more. P1, difference between two gait speed groups; P2, gender difference.

Individuals with SGS had lower education levels, more diseases (15% more), a higher rate of polypharmacy (over 10% more), and spent approximately twice as much money on healthcare as people in the NGS group. The mean medical costs for men and women in the SGS group were 9,778 yuan (RMB) and 5,893 yuan (RMB), compared to 4,210 yuan (RMB) and 3,196 yuan (RMB), respectively, in the NGS group (*p* < 0.0001). Compared with the NGS group, there were more people in the SGS group needing care (about 10% more) due to illness and hospitalization (about 20% more of the males and 10% more of the females) during the preceding year. Additionally, more (about 10% more) SGS individuals were dissatisfied with their health, had lower MMSE scores.

**Figure 1:**
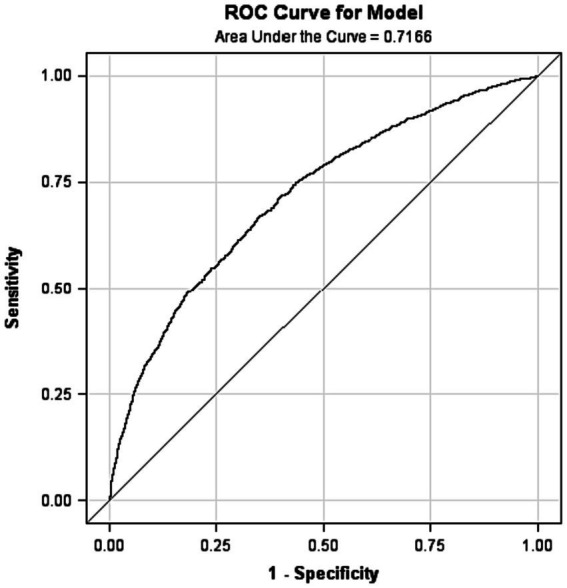
Receiver-operator characteristic (ROC) curve for the probability of slow gait speed produced by logistic regression model.

**Figure 2:**
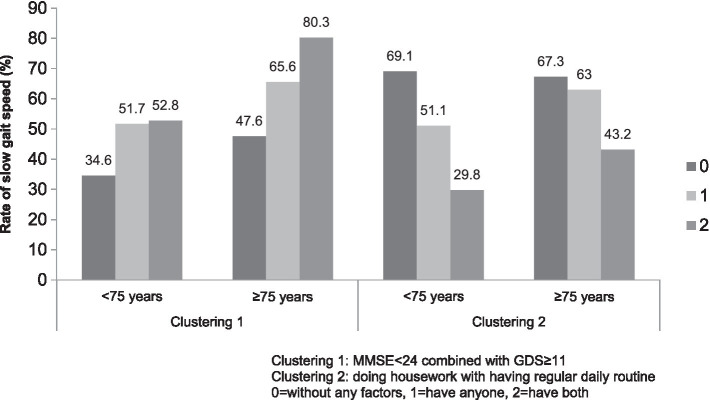
The number of factors clustering with the risk of slow gait speed.

The stepwise logistic regression model (Table 2) revealed that co-morbidity (*OR* = 1.81, 1.58–2.07), polypharmacy (*OR* = 1.47, 1.25–1.74), MMSE <24 (*OR* = 1.85, 1.54–2.22), and GDS ≥ 11 (*OR* = 1.40, 1.18–1.65) were associated with SGS. Conversely, higher education level (*OR* = 0.77, 0.67–0.88), DHW (*OR* = 0.43, 0.38–0.49), having a RDR (*OR* = 0.64, 0.45–0.91), and current alcohol consumption (*OR* = 0.74, 0.62–0.90) were inversely associated with SGS. On the other hand, the logistic regression model did not reveal any association between age, gender, current smoking, physical exercise, dissatisfaction with personal health, and disinterest in life and SGS. The AUC value on ROC analysis was 0.72 for the selected variables (Figure 1). With the clustering of MMSE<24 and/or GDS ≥ 11(0 = none, 1 = having either one, 2 = having both factors), the risk of SGS gradually increased with increasing cluster score, while with increased clustering score of DHW and/or RDR (0 = none, 1 = having either one, 2 = having both factors), the risk of SGS was reduced (Model 2). Figure 2 shows the pattern of change in the risk of SGS with the increasing clustering score of MMSE <24 with GDS ≥ 11 and of increasing clustering score of DHW with RDR. The increasing risk of SGS with the clustering number of MMSE <24 and GDS ≥ 11 are more apparent in people aged≥75. On the other hand, DHW clustering with RDR greatly reduces the risk of SGS in people aged <75, and the benefit is evident even in people aged≥75.

**Table 2:** Factors associated with slow gait speed by logistic regression.

Factors	*β*	SE	Wald χ^2^	*p-*value	*OR* (95% CI)
Model 1					
Intercept	0.6813	0.2069	10.8418	0.0010	–
Education≥12 years	−0.2681	0.0692	15.0137	0.0001	0.77 (0.67–0.88)
Co-morbidity	0.5905	0.0691	72.9677	<0.0001	1.81 (1.58–2.07)
Polypharmacy	0.3865	0.0842	21.0553	<0.0001	1.47 (1.25–1.74)
MMSE scores <24	0.6150	0.0926	44.0920	<0.0001	1.85 (1.54–2.22)
GDS scores≥11	0.3342	0.0859	15.1287	0.0001	1.40 (1.18–1.65)
Doing housework	−0.8533	0.0657	168.4215	<0.0001	0.43 (0.38–0.49)
RDR	−0.4452	0.1774	6.2962	0.0121	0.64 (0.45–0.91)
Current drinker	−0.2974	0.0962	9.5528	0.0020	0.74 (0.62–0.90)
Model 2*					
Clustering 1	0.5055	0.0790	40.9818	<0.0001	1.66 (1.42–1.94)
Clustering2	0.8208	0.1464	31.4269	<0.0001	2.27 (1.71–3.03)
Clustering 3	−0.3991	0.2057	3.7625	0.0524	0.67 (0.45–1.00)
Clustering4	−1.2402	0.2074	35.7522	<0.0001	0.29 (0.19–0.43)

Co-morbidity: having ≥ two common chronic diseases. Polypharmacy: taking ≥ five kinds of medicines. MMSE: Mini-mental State Examination. GDS: Geriatric Depression Scale. RDR: Regular daily routine:1 = unstructured, 2 = structured. Current smoking: ≥five cigarettes/day. Current drinking: at least once a week. In Model 1, age, gender, current smoking, and physical exercise, dissatisfaction in health, and disinterested in life were not selected by logistic model. *Model 2 only shows the results of variable clusterings. Clustering1: having MMSE scores < 24 or GDS scores ≥ 11. Clustering2: having both MMSE scores < 24 and GDS scores ≥ 11.Clustering3: doing housework or having regular daily routine. Clustering4: doing housework and having regular daily routine.

## Discussion

The data from this study showed that reduced physical function implies an overall reduction, including mental and physical, in health status in older Chinese individuals (10). As a result, the medical cost in the SGS group was much higher than that in the NGS group. GS is a quick, inexpensive, and useful indicator of health in older adults who have chronic and expensive declines in health that might be circumvented by targeted intervention (11–13). Our study identified a strong association between DHW (Table 2) (*OR* = 0.43, 0.38–0.49) and RDR (Table 2) (*OR* = 0.64, 0.45–0.91) with physical function. This result confirms our hypothesis that old persons’ active daily life engagement and daily routine will benefit their physical performance (14). The individual’s own motivation to respond to the challenges of aging should be encouraged (5). The effects of clustering DHW and RDR on the risk of SGS were also studied. The data showed that active engagement in daily life (represented by DHW) plus a RDR will have a greater benefit on the physical function in the older people (*OR* = 0.29, 0.19–0.43) (Table 2) than either factor alone. The gain in physical function was significant even in the people aged≥75 (Figure 2). The clustering of the two factors gives us a picture to describe the older people’s daily life and reveal an addictive effect on their functional well-being.

Regular PE is often considered an indicator of a healthy lifestyle and contributes to physical well-being (2). Instead of PE, DHW was selected by the logistic model and showed the strongest inverse association with SGS. The reason PE did not stand out may be explained by the similarly high rate and intensity of PE observed in both the SGS and NGS groups (Table 1). DHW is an unstructured daily life activity that requires mild-to-moderate physical and intellectual engagement. Previous studies have shown that energy expenditure doing housework represent 35.2% of the total activity in subjects aged 65–74 (15). Other studies have shown that the time devoted to housework activities can be equivalent to the time spent on moderate-intensity activity (16). For a population of retired individuals, energy expended doing housework makes up, to some degree, for the reduction in energy that they had previously expended through their employment. The result suggests that DHW may help older people maintain their physical function.

For older people, DHW could also reflect the ability to live independently and their desire to make contributions to family development, which is consistent with the concept of aging well (6). Studies have shown that older adults who are more autonomous in their daily life and environment are more likely to experience happiness (17, 18). Additionally, a longitudinal study among older adults in Australia reported that DHW could maintain the mental function of older individuals and reduce the risk of dementia. Similar studies have also shown that non-sedentary older individuals present fewer indications of depression and dementia (19), which are both essential factors for maintaining independence and autonomy of life. In summary, based on previous studies and the strong association of DHW with faster GS in our study, there is ample evidence that daily life engagement is associated with multiple benefits, such as physical, cognitive, and mental well-being in older populations.

The benefit of RDR on health status has not been as fully noted. In this study, RDR was found to have an inverse association with SGS. The presence of RDR among retired individuals may reflect their positive motivation to maintain their health and lead to having a regular bio-clock, resulting in benefits to their functional well-being. This finding supports the idea that RDR may be an under-recognized modifiable factor that could benefit physical performance, including activities of daily living and more vigorous activities like climbing stairs (20). Additionally, RDR may play a role in the development of circadian rhythm, and research has explored the association between circadian rhythm and GS, albeit in a limited sample of older individuals with stroke (21). While these findings suggest a potential association between circadian rhythm and GS, further comprehensive studies are necessary to explore this relationship more extensively.

The further analysis of the factors associated with the risk of SGS in our population revealed that as expected, co-morbidity, polypharmacy, MMSE <24, and GDS ≥ 11 were associated with the risk of SGS (10, 22–26). The association of polypharmacy with SGS suggests that medical treatment did not result in improved physical functions (27, 28). Very commonly, impaired cognitive function and symptoms of depression commonly co-exist in older populations (29) and are associated with a decline of physical function (30, 31). Our study revealed that the clustering of these symptoms result in much higher risk of decline in physical function (*OR* = 2.27, 1.71–3.03, Table 2), particularly in individuals aged ≥75 (Figure 2). This evidence suggest that psychology should receive greater attention in efforts to improve the functional well-being of older individuals (32). In short, the co-exiting and mutual association of the cluster of low physical performance, cognitive function, and depressed mood suggest that multiple benefits could be gained through effective intervention (32).

Similar to the findings reported in the study by Woo and colleagues of a population of older Chinese in Hong Kong, which found that alcohol intake was associated with faster walking speeds. In both studies, most of the drinkers were males, so very likely drinking takes place in social activities. If this is true, then it may be the social atmosphere in which the drinking takes place in social activities that lead to an improved psychological state, rather than the drinking itself, which may help drinkers maintain faster walking speeds (33).

GS is considered a comprehensive index of physical function that reflects the overall health status of an individual and serves as a marker of physiological reserve (13, 22, 23, 33). Woo et al. found that a fast-walking speed (>1.39 m/s), the opposite of SGS, maybe a sign of successful aging, since the variables significantly associated with fast walking are similar to the attributes of successful aging, and fast walkers exhibit a robustness and avoidance of frailty (33). In our study, the difference between the mean GS of the slow and normal GS groups for each gender was about 0.5 m/s (1.0 m/s vs. 0.5 m/s). A change in GS of as little as 0.1 m/s has an observable effect on health. Specifically, study show that a decline in gait speed of 0.1 m/s within 1 year increased the subsequent 5-year mortality rate (34). In contrast, a GS increase of 0.1 m/s has been associated with observable health benefits, e.g., an increase as a result of intervention has been associated with a 17.7% reduction in the absolute risk of death (35), fewer hospitalization days (2.3 fewer days), and 1-year cost reduction of $1,188 (36). Based on this evidence, it can be expected that improving engagement in daily life, having a regular lifestyle, and engaging in more social activities may bring result in appreciable gains in physical function among older individuals. However, the underlying mechanisms and their impacts on the trajectory of function changes require further study.

This study has some limitations. Firstly, as a cross-sectional study, it is difficult to establish a cause-effect relationship between the studied factors and poor physical function. Secondly, the intensity and type of housework are not clearly measured, so specific suggestions could not be further evaluated in this study. Thirdly, GS was evaluated by measuring the time for the subject to walk 20 m (waking 10-meter plus a U-turn back to starting line), which is not often used for measuring GS. Future studies will aim to address these limitations, especially by carrying out a cohort study to validate the association of daily life engagement with physical functions in the older people.

## Conclusion

The evidence from this study supports the importance of encouraging older people to keep daily life engagement and have a regular routine, which will benefit them in maintaining their physical function.

## Data availability statement

The raw data supporting the conclusions of this article will be made available by the authors, without undue reservation.

## Ethics statement

The studies involving humans were approved by the Ethics Committee of Beijing Hospital, Ministry of Health. The studies were conducted in accordance with the local legislation and institutional requirements. The participants provided their written informed consent to participate in this study. Written informed consent was obtained from the individual(s) for the publication of any potentially identifiable images or data included in this article.

## Author contributions

RL: Writing – original draft, Data curation. YD: Data curation, Writing – original draft. YH: Data curation, Investigation, Conceptualization, Writing – review & editing. CZ: Writing – review & editing. JP: Data curation, Investigation, Conceptualization, Writing – review & editing. JL: Funding acquisition, Software, Writing – review & editing. TZ: Funding acquisition, Project administration, Validation, Writing – review & editing. PZ: Funding acquisition, Validation, Writing – original draft, Writing – review & editing.
